# Screening of household contacts for TB infection in Cote d'Ivoire

**DOI:** 10.5588/ijtldopen.23.0342

**Published:** 2024-01-01

**Authors:** R. K. N’Guessan, D. A. B. Orsot, J.M. Ahui Brou, N. K. Bamba, M. E. Tchoutedjem Mefo, A. S. Bakayoko

**Affiliations:** ^1^ Unité des Mycobactéries Tuberculeuses et Atypiques, Laboratoire National de Référence de la Tuberculose, Institut Pasteur de Côte d'Ivoire, Abidjan,; ^2^ Unité de formation et de recherche (UFR) des Sciences Médicales Université Felix Houphouët-Boigny Service de Pneumologie Centre Hospitalière Universitaire (CHU) de Cocody,; ^3^ Programme National de Lutte contre la Tuberculose Abidjan; ^4^ UFR des Sciences Médicales Université Felix Houphouët-Boigny Service de Pneumologie CHU de Treichville, Abidjan, Côte d'Ivoire

**Keywords:** latent tuberculosis infection, screening, costs, QALYs

## Abstract

**SETTING:**

Côte d'Ivoire is a country with a high incidence of TB. The control of TB infection is focused on high-risk patients but has limited implementation.

**OBJECTIVE:**

Cost-benefit analysis of TB infection (TBI) screening of household contacts in Côte d’Ivoire to evaluate economic implications of the implementation of interferon-gamma release assays (IGRAs) and the tuberculin skin test (TST).

**DESIGN:**

We compared the effectiveness of QuantiFERON-TB Gold Plus (QuantiFERON) with the TST using an economic model previously evaluated in medium TB incidence settings. Principal outcomes relating to TBI screening, as well as the lifetime costs and benefits of the patient cohort, were captured using a decision tree, followed by a Markov model.

**RESULTS:**

QuantiFERON proved to be both more effective and less costly than TST. Compared to QuantiFERON, TST use leads to an approximate 33% increase in the lifetime risk of developing active TB.

**CONCLUSIONS:**

For household contacts of active TB cases in Côte d’Ivoire, QuantiFERON is cost-effective when compared with TST. *R* shiny interactive interface enables model customisation for different scenarios, settings, risk groups and TBI screening methods. Further research should be conducted in similar settings to generalise the results.

## RÉSUMÉ

**CONTEXTE:**

La Côte d'Ivoire est un pays où l'incidence de la TB est élevée. La lutte contre l'infection à TB est axée sur les patients à haut risque, mais sa mise en œuvre est limitée.

**OBJECTIF:**

Analyse coût-bénéfice du dépistage de l'infection à TB (TBI) chez les contacts familiaux en Côte d'Ivoire afin d'évaluer les implications économiques de la mise en œuvre des tests de libération de l'interféron-gamma (IGRA) et du test cutané à la tuberculine (TST).

**DESIGN:**

Nous avons comparé l'efficacité de QuantiFERON-TB Gold Plus (QuantiFERON) avec celle du TST en utilisant un modèle économique précédemment évalué dans des contextes d'incidence moyenne de la TB. Les principaux résultats relatifs au dépistage de la TBI, ainsi que les coûts et bénéfices à vie de la cohorte de patients, ont été saisis à l'aide d'un arbre de décision, suivi d'un modèle de Markov.

**RÉSULTATS:**

QuantiFERON s'est avéré à la fois plus efficace et moins coûteux que le TST. Par rapport à QuantiFERON, l'utilisation du TST entraîne une augmentation d'environ 33% du risque de développer une TB active au cours de la vie.

**CONCLUSIONS:**

Pour les contacts familiaux des cas de TB active en Côte d'Ivoire, QuantiFERON est rentable par rapport au TST. L'interface interactive *R* shiny permet de personnaliser le modèle pour différents scénarios, contextes, groupes à risque et méthodes de dépistage de la TBI. D'autres recherches devraient être menées dans des contextes similaires pour généraliser les résultats.

According to the WHO, TB is a significant cause of mortality, causing around 1.5 million deaths annually worldwide, ranking among the top 10 preventable causes of death.^1^ Approximately 1.7 billion individuals, a quarter of the global population, are estimated to be infected with TB-causing *Mycobacterium tuberculosis*.^2^ Most cases remain asymptomatic, while 10% may progress to active TB. The COVID-19 pandemic impacted TB services, including TB infection (TBI) screening and prevention, hindering progress toward the End TB Strategy goals achieved by 2019.^1^

Timely diagnosis and treatment of TBI to prevent TB transmission and the consequent replenishment of the TBI reservoir are therefore essential for achieving TB elimination.^3,4^ TBI can occur upon contact with individuals who have active TB. The risk of TBI is particularly high in conditions involving close and prolonged exposure with an infectious individual. Therefore, the household contacts of cases with transmissible forms of TB is the population group with the highest risk of being infected with TB and develop the disease.^5^

Systematic TBI testing and treatment have been shown to be beneficial in scenarios where the likelihood of exposure to TB disease is high including household contacts.^6,7^ These studies, including historical ones showing a decline of TB incidence up to 17% per year^8^ revealed that early detection and treatment of TBI will result in a significant decrease in incident TB cases among contacts. Based on available evidence, the WHO guidelines provide strong recommendations for systematic screening, using both interferon-gamma releasing assays (IGRA) and tuberculin skin tests (TSTs), and treatment of household contacts of TB cases in all settings regardless of TB incidence and/or resources availability emphasising the benefits of early detection and treatment of TBI to prevent the spread of TB within households and communities.^7^

The infrastructure and personnel qualifications required for the use of IGRAs are viewed as significant obstacles that impede the successful implementation of IGRAs in low- and middle-income countries (LMICs). Moreover, due to several limitations of the TST, such as low specificity, difficult administration and ongoing global supply shortages, there is an increasing consensus in favour of offering preventive therapy as a potential solution in LMICs.^9^ However, this approach, seemingly simplifying the diagnostic process and eliminating the need for expensive tests such as IGRAs or repeated TSTs, has major limitations associated with indiscriminate use of preventive therapy, potentially leading to unnecessary medication usage, acquisition of drug resistance and the diversion of resources from other critical healthcare needs. In a recent systematic review, prevalence of TBI in high TB incidence countries, including India and sub-Saharan Africa was estimated at 26.6–36.0%, strongly supporting the test-and-treat approach, i.e., screening for TBI, followed by treatment of eligible individuals.^10^

Côte d’Ivoire is classified as a high TB burden country, with an estimated 128 cases/100,000 population.^1^ The TB-HIV co-infection rate is 17 cases/100,000, and the multidrug-resistant/rifampicin-resistant TB (MDR/RR-TB) rate is 5.7/100,000.^1^ Bacilli Calmette-Guérin (BCG) vaccination coverage is at 93%.^1^ National guidelines of Côte d'Ivoire recommend TST for confirming TBI among household contacts, including children up to 5 years old. TST implementation is limited. The guidelines suggest treating children under 5 and HIV patients with isoniazid (INH) for 6 months. In 2022, 6,104 TBI cases were detected, with 6,001 in children under 5.^1^

IGRA is a blood test that detects the immune response to *M. tuberculosis*-specific antigens. The WHO has endorsed three IGRAs, the T-SPOT.*TB* test (Oxford Immunotec, Abingdon, UK) and QuantiFERON-TB Gold Plus (QuantiFERON; Qiagen, Hilden, Germany) being the two most widely used.^11^ Both IGRAs have been shown to have a higher sensitivity than TST.^12^ A comprehensive analysis of multiple studies revealed that IGRAs consistently exhibit high levels of specificity, while the specificity of TST is low and varies significantly in BCG-vaccinated populations.^13^ The test requires a single visit for a blood draw, and no patient return visits are required for reading the test.^14^ Furthermore, as the test is carried out in a laboratory setting, it yields objective and qualitative results with a unique cut-off for determining positive and negatives results.

High costs are considered among the key barriers to IGRA implementation in LMICs.^15^ In addition to evaluating technical aspects, a crucial component employed by healthcare decision-makers is the inclusion of cost-effectiveness analyses, which involves comparing the health effects of two interventions in relation to their associated costs.

At present, it remains uncertain which of the existing TBI screening tests is the most cost-effective approach for screening household contacts in high TB burden settings such as Côte d’Ivoire. The aim of the present study was to estimate the potential cost-effectiveness of QuantiFERON in comparison with other TBI screening options in household contacts. The results of this study aim to guide policy makers in Côte d’Ivoire, as well as other countries facing similar challenges.

## METHODS

### Model overview

The economic model, based on R v0.2 (R Computing, Vienna, Austria) with an interactive Shiny interface, has previously been described by Barker et al. and customised to compare the effectiveness of QuantiFERON-TB Gold Plus (QuantiFERON) with TST from a healthcare perspective.^16^ The model comprised active TB screening conducted prior to TBI screening, wherein patient screening was accomplished using either QuantiFERON or TST. Positive results led to TBI treatment and potential non-adherence. In the Markov model, health states, transitions and TBI reactivation were taken into account; it was assumed that successful TB treatment would eliminate the risk of reactivation (Table 1).

**Table 1.** Transition probabilities

From	Healthy %	Untreated TBI (treatment-naïve) %	TBI, taking treatment without AE %	TBI, taking treatment with AE %	Untreated TBI (previously-treated) %	DS-TB being treated %	MDR-TB being treated %
Healthy	100	0	0	0	0	0	0
Untreated TBI (treatment-naïve)	0	Residual*	0	0	0	Calculated from TBI reactivation rates*	Calculated from TBI reactivation rates*
TBI, taking treatment without AE	Calculated from treatment effectiveness	0	0	0	Calculated from treatment effectiveness	0	0
TBI, taking treatment with AE	Calculated from treatment effectiveness	0	0	0	Calculated from treatment effectiveness	0	0
Untreated TBI (previously-treated)	0	0	0	0	Residual*	Calculated from TBI reactivation rates*	Calculated from TBI reactivation rates*
DS-TB being treated	100	0	0%	0%	0%	0%	0%
MDR-TB being treated	100	0	0%	0%	0%	0%	0%

* Values were updated every cycle.

AE = adverse event; TBI = tuberculosis infection; DS-TB = drug-susceptible TB; MDR-TB = multidrug-resistant TB.

The model presented the lifetime rate of developing active TB (drug-susceptible and multidrug-resistant TB) per patient in the cohort, along with the incremental difference between TST and QuantiFERON. This difference, along with the incremental costs, was used to calculate the additional costs of treating one active TB case with QuantiFERON compared to TST. The number needed to treat (NNT) to prevent one additional case of active TB using QuantiFERON compared to TST was also reported.

The incremental cost-effectiveness ratio was used to measure cost-effectiveness, with a conservative threshold of 0.52 times Côte d'Ivoire's gross domestic product per capita (US$2,549). Both costs and benefits were discounted at 3% annually.^17^ Outcomes included cost per quality-adjusted life-year (QALYs) gained and TB cases averted. QALYs were accumulated throughout the model, representing health-related quality of life in each state.

### Ethics statement

As no patient-level data were used, ethical approval was not required.

### Model parameters

Model parameters and their sources are provided in Table 2. We assumed that patients with TBI received 6-month daily INH treatment as per the WHO TBI guidelines.^7^ The baseline inputs for adherence, adverse events and treatment efficacy were based on this 6-month INH regimen.^7^ Screen test sensitivity and specificity rates were sourced from previous research.^13,18^ The model also included a parameter to calculate a weighted average of TST specificity based on the proportion of BCG-vaccinated individuals in the cohort.

**Table 2.** Model inputs

Parameter	Value	Standard error	PSA distribution	Reference
Population size	376,181	N/A	N/A	2
Average age	18.9	N/A	N/A	3
Proportion male	0.521	N/A	N/A	32
TBI prevalence	0.202	0.020	Beta	4
All-cause mortality	Age dependant	N/A	N/A	5
DS-TB mortality risk	0.125	0.0125	Beta	6
MDR-TB mortality risk	0.375	0.0375	Beta	6
Treatment efficacy	0.69	0.069	Beta	7
Adherence rate	0.67	0.067	Beta	Country-based data (assumes 6-month daily isoniazid treatment)
Probability of AE (drug-related hepatotoxicity)	0.36	0.036	Beta	
Initial reactivation rate (treatment-naive)	0.0333	0.0033	Beta	8
Initial reactivation rate (previously treated)	0.073	0.0073	Beta	8
Proportion of MDR-TB cases (treatment-naive)	0.046	0.0046	Beta	9
Proportion of MDR-TB cases (previously treated)	0.022	0.0022	Beta	
Proportion BCG-vaccinated	0.93	N/A	N/A	32
Sensitivity of TST	0.77	0.03	Beta	10
Specificity of TST (BCG-vaccinated)	0.59	0.07	Beta	
Specificity of TST (non-BCG-vaccinated)	0.97	0.03	Beta	
Sensitivity of QuantiFERON	0.91	0.03	Beta	11
Specificity of QuantiFERON	0.95	0.01	Beta	
Utility values				
Healthy	1	N/A	N/A	12, 13
Untreated TBI	1	N/A	N/A	
TBI, taking treatment without AE	0.99	0.099	Beta	
TBI, taking treatment with AE	0.85	0.085	Beta	
DS-TB	0.8	0.08	Beta	
MDR-TB	0.58	0.058	Beta	

PSA = probabilistic sensitivity analysis; N/A = not available; TBI = TB infection; DS-TB = drug-susceptible TB; MDR-TB = multidrug-resistant TB; AE = adverse event; BCG = bacille Calmette–Guérin; TST = tuberculin skin test.

The model included an age-dependent rate of all-cause mortality based on the average age and proportion of males in the patient population, based on Côte d'Ivoire life tables.^19^ TBI was assumed to pose no additional mortality risk, while mortality risk due to active TB and adverse events (AEs) were included in the model, with the latter set to 0% in the base case due to limited evidence. The proportion of MDR-TB cases was derived from a recent national drug resistance survey.^20^

Unless otherwise mentioned, national sources for Côte d'Ivoire were primarily used to obtain the unit costs and resource use information. Resource use data (Table 3) were combined with unit costs (Table 4) to determine the total estimated costs of screening and management of TB. In decision trees, particular resources were expended once per branch, whereas the Markov model calculated resource utilisation per cycle, with patients utilising resources continuously during their duration in each health state.

**Table 3.** Resource use*

Resource	TBI without AE	TBI with AE	Drug-susceptible TB	Multidrug-resistant TB
Healthcare worker contact time, h				
Nurse time	1	1.25	1.3	157.5
General practitioner time	0	0	0.15	0.15
Hospital physician time	0	0.25	0	0
TB specialist time	0	0.25	1.3	2.63
DOT sessions	0	0	74.7	0
Diagnostic tests, *n*				
Sputum smear tests	0	0	5	13
Sputum cultures	0	0	2	16
Chest X-rays	3	6	4.38	6.6
ESR	0	0	2.15	6.4
GeneXpert (PCR test)	0	0	1	1
Laboratory time for diagnosis tests, h	1	2	3	12
AEs				
Liver function test, *n*	0	2	0.46	2.4
Laboratory time for liver function test, h	0	2	0.5	2
Treatment of drug-induced hepatotoxicity, *n*	0	2	0.46	1.2
Active TB case contacts				
Contacts per active case, *n*	0	0	3	3
Other				
Hospitalisation, days	0	14	6.9	162

* All resource use were sourced by QIAGEN from national sources for Malaysia. The resource values in the PSA were assumed to have a standard error of 10% and a Gamma distribution was used.

TBI = TB infection; AE = adverse event; DOT = directly observed therapy; ESR = erythrocyte sedimentation rate; PCR = polymerase chain reaction; PSA = probabilistic sensitivity analysis.

**Table 4.** Unit costs

Parameter	Description	Cost (US$)	Source
TBI treatment	The average cost of TBI treatment per patient	49.5	GOF
DS-TB treatment	The average cost of treating DS - TB per patient	68.9	GDF
MDR-TB treatment	The average cost of treating MDR - TB per patient	927.38	GDF
QuantiFERON	The cost of a QuantiFERON test for one patient	15.9	GDF
TST	The cost of a TST for one patient	2.4	Country
Healthcare worker contact time			
Nurse time	The cost of a nurse per hour	1.74	Country
Hospital physician lime	The cost of a hospital physician per hour	2.93	Country
General practitioner	The cost of a general practitioner per hour	2.93	Country
TB specialist time	The cost of a TB specialist per hour	2.13	Country
DOT sessions	The cost of a DOT session	1.74	Country
Environmental officer	The cost of an environmental health officer per hour	1.19	Country
Diagnostic tests			
Smear test of sputum	The cost of a smear lest per patient	0.32	GDF
Culture of sputum examination	The cost of a culture test per patient	6.7	GDF
Chest X - ray	The cost of a chest X-ray per patient	8.41	Country
ESR	The cost of an ESR per patient	5.05	Country
GeneXpert ( PCR test )	The cost of a GeneXpert tests to check for drug sensitivity per patient	9.98	GDF
Laboratory technician time	The cost of a laboratory technician per hour	1,74	Country
Adverse events			
Liver function test	The number of liver function tests per patient	6.73	Country
Laboratory technician time	The cost of a laboratory technician per hour	1.74	Country
Treatment of drug - induced hepatotoxicity	The average cost of treatments per patient	7.03	14
Active TB case contacts			
Contacts per active case	The average cost associated with contacts per active case of TB	25.09	Country
Other			
Hospitalisation	The average cost of hospitalisation per day	3.6	Country

TBI = TB infection; GDF = Global Drug Facility; DS-TB = drug-susceptible TB; MDR-TB = multidrug-resistant TB; TST = tuberculin skin test; DOT = directly observed therapy; ESR = erythrocyte sedimentation rate; PCR = polymerase chain reaction; PSA = probabilistic sensitivity analysis.

### Sensitivity analysis

A deterministic sensitivity analysis (DSA) was conducted to assess the first-order uncertainty surrounding the input parameters of the model. Model results were evaluated using monetary benefit, which was determined by multiplying the total QALYs generated by each screening method with the cost-effectiveness threshold and subtracting the total cost. Upper and lower bounds were applied to each parameter, representing a 15% increase and decrease from the base analysis estimates. A probabilistic sensitivity analysis (PSA), involving 5,000 iterations with varied input values drawn from their distributions, was also performed. Model convergence was visually assessed, and results were displayed on a cost-effectiveness graph and assessed for cost-effectiveness acceptability.

In addition to the DSA and PSA, three scenario analyses were conducted. The first scenario explored the potential impact of improved treatment adherence rates, increasing them from 67% to 85% for TBI treatment with INH. The second scenario investigated the effect of enhanced treatment efficacy, increasing base case efficacy from 69% to 93% for the 6-month INH treatment. The third scenario examined the consequences of increased TBI prevalence and active TB reactivation rates. In this scenario, TBI prevalence was raised to 34%, and reactivation rates were adjusted, providing valuable insights into their influence on cost-effectiveness outcomes.

## RESULTS

Overall costs per individual were US$45.33 for TST and US$38.16 for QFT. QFT had higher screening costs (US$17.21 vs. US$3.87 for TST), but compared to TST, QFT had lower treatment costs (US$7.96 vs. US$16.02), healthcare worker costs (US$1.07 vs. US$1.69), TBI AE costs (US$1.71 vs. US$3.50), diagnosis test costs (US$6.87 vs. US$13.81), active TB case costs (US$0.38 vs. US$0.51) and other associated costs (US$2.97 vs. US$5.94).

The individual patient case results are shown in Table 5. Over a lifetime horizon, QuantiFERON is the dominant strategy approach, being both more effective and less costly than TST. Therefore, QuantiFERON is considered cost-effective when compared to TST. Compared with QuantiFERON, the lifetime risk of developing active TB increases by approximately 33% for TST. This means that compared to TST, QuantiFERON screening reduces the likelihood of individuals developing active TB when receiving TB preventive therapy (TPT). When compared with QuantiFERON, TST leads to an incremental 220 active TB cases/100,000 individuals. Conversely, the screening threshold required to avert a single additional case of active TB, in comparison to QuantiFERON, is 456.

**Table 5.** Base case results per patient

Strategy	Cost US$	QALY	Change in costs US$	Change in QALY_S_	ICER (cost per QALY gained)
QuantiFERON	38.16	27.07			
TST	45.33	27.05	$ 7.17	-0.02	Dominated

QALY = quality-adjusted life-year; ICER = incremental cost-effectiveness ratio; TST = tuberculin skin test.

### Sensitivity analysis

In the DSA, the discount rate for benefits is identified as the main factor impacting the economic evaluation. Additional supplementary documents include graphical illustrations of the 10 most significant drivers of the economic model based on DSA. However, the results of the DSA did not alter the decision outcome across all instances. The PSA shows that QuantiFERON is cost-effective compared to TST in 100% of the simulations, regardless of the chosen willingness-to-pay threshold. Furthermore, in every scenario analysed, QuantiFERON proves to be the cost-effective (dominant) strategy in comparison to TST.

## DISCUSSION

The results suggest that QuantiFERON is cost-effective when compared with TST for use in household contacts in Côte d’Ivoire. Furthermore, this study emphasises the need for a comprehensive cost-effectiveness analysis to uncover hidden costs. While the initial QFT screening of TBI patients was 4.4 times more expensive than TST (US$17.21 vs. US$3.87, respectively), it is important to assess the broader financial context. Our analysis revealed that the subsequent costs associated with treatment, AEs, additional diagnostic tests and other related expenses heavily influenced the overall economic evaluation. Specifically, QFT resulted in treatment costs that were nearly half of those incurred by TST. Healthcare worker expenses related to QFT were approximately 63% of those for TST. TBI AE costs for QFT were roughly half of TSTs, and the diagnostic expenses for QFT were about 50% of those for TST. Further, the costs of active TB cases were marginally less for QFT than for TST, and other related expenses for QFT were around 50% of those for TST. When we combine these factors, the overall costs per individual amount to US$45.33 for TST and US$38.16 for QFT. This demonstrates that, despite the higher initial screening cost, QFT presents significant economic advantages in the medium- and long-term.

In the base case scenario, the probability of QuantiFERON being the most cost-effective option compared to TST was approximately 100%. Despite uncertainties in model inputs and assumptions, the sensitivity and scenario analyses confirm the model's robustness.

Regardless of any fluctuations in the top 10 drivers of the economic model, the direction of the cost-effectiveness outcomes remained consistent. However, the DSA results underscored the fact that the reactivation rate of TB and the TBI prevalence had a substantial impact on the results.^21,22^ In this study, the prevalence of TBI could potentially exceed our initial estimation.^23^ Drawing upon global burden database, the TBI prevalence in Côte d’Ivoire was found to be 20.2%.^24^ Regardless of whether TST or IGRA detection methods were used to estimate the TBI prevalence, a meta-analysis indicates a comparable worldwide prevalence ranging from 21.2% to 24.8%.^24^ Despite the mentioned uncertainties, in a hypothetical scenario involving increased TBI prevalence and a higher TB reactivation rate, the analysis revealed that QuantiFERON was the cost-effective option in comparison to TST.

Considering the lower sensitivity of TST compared to QuantiFERON, a greater number of false-negatives occurred, leading to a larger number of individuals progressing to active TB over lifetime horizon. Consequently, this contributed to a reduction in QALYs. Similarly, Côte d’Ivoire has a high proportion of individuals who have received BCG vaccination. This is of particular importance when considering the TST for TB screening, as TST specificity is known to be poor in vaccinated populations, leading to an increased likelihood of false-positives in such settings.^25^ These false-positives not only inflate the apparent prevalence of TBI, but also lead to additional downstream costs. Such costs might include unnecessary follow-up tests, consultations and treatments. Moreover, an increase in the number of patients receiving unnecessary TPT increases the likelihood of treatment-related AEs, ultimately resulting in a decrease in QALYs.

Overall, our analysis confirmed previous findings that while the unit cost of TST is cheaper than that of QFT, a poorer performance of TST incurs additional expenses that ultimately translate into a more expensive solution.^16,26^ Recent advancements, such as the approval of novel antigen-based skin tests such as Diaskintest (Generium, Moscow, Russian Federation), C-Tb (Serum Institute of India, Pune, India) and C-TST (Anhui Zhifei Longcom, Anhui, China) by the WHO, aim to address some of the limitations associated with TST.^27^ However, these tests, although offering a promising alternative to TST, come with their own set of challenges. Operationally, they share many of the TST’s limitations, including the need for specialised training for administration and reading of results, quality control challenges, the possibility of subjective interpretations, and the requirement for a return visit to read the results, cold chain for storage and transportation of antigen preparations. In addition, evidence on the cost-effectiveness of novel skin tests remains extremely limited, warranting a comprehensive analysis vs. IGRA and TST that takes into account both direct and indirect costs. Screening household contacts for TBI is crucial, as per WHO recommendations, to work toward achieving the goals of the End TB Strategy and progressing toward TB pre-elimination.^4,5^ By identifying TBI among household contacts, TPT can be initiated, reducing the risk of progression to active TB. This approach plays a significant role in breaking the chain of transmission within households and communities.

Multiple publications have demonstrated the cost-effectiveness of TBI screening among household contacts, resulting in improved health outcomes and reduced healthcare costs.^26^ Studies have shown that identifying and treating TBI in this population is a worthwhile investment, resulting in improved health outcomes and reduced healthcare costs.^26^ These findings reinforce the importance of implementing TBI screening programmes for household contacts to effectively control TB and allocate healthcare resources efficiently. However, direct comparison of studies is challenging due to variations in modelling approaches, which include factors such as the exclusion of active TB cases and differences in the treatment regimens used.

The study's model had several strengths, offering high flexibility to end-users, and permits the customisation of inputs, allowing for the exploration of different TBI assays, scenarios and settings. The model dynamically updates results, facilitating a comprehensive analysis of various parameters in TBI contexts. Its design provides fine-grained control over cost and resource parameters, aligning with specific requirements and WHO recommendations, making it applicable for robust analysis and decision-making.

Our study had several limitations. First, the limited availability of TBI data specific to household contacts in Côte d’Ivoire meant that some parameters had to be sourced from studies conducted in similar settings. This could potentially impact the estimated prevalence of TB or the reactivation rate, as multiple risk factors can influence TB risk.^28^

Second, the availability of multiple treatments for TB management^7^ led to the model’s assumption of one treatment regimen per TB status, predominantly used in Côte d'Ivoire. Although alternative regimens may affect adherence, AEs and cost, the model's flexibility permits users to explore different regimens and analyse their potential impacts by adjusting associated inputs.

Despite the inherent limitations of the model, the sensitivity analysis confirmed its robustness. Notably, the analysis revealed that the decision outcomes remained consistent, underscoring the model's resilience to the assumptions and limitations outlined.^29–31^ These findings provide assurance that the model can withstand uncertainties and variations in its inputs, lending credibility to its overall reliability and applicability in informing decision-making processes.

## CONCLUSION

This study, aimed at assessing the cost-effectiveness of TBI screening for household contact in Côte d’Ivoire, has demonstrated that QuantiFERON is a cost-effective option compared to the TST. Future research should focus on exploring the cost-effectiveness of TBI screening strategies for household contacts in other high TB burden countries, and thereby, contribute to accelerate TB elimination.
